# Molecular Mechanisms of Cutaneous Immune-Related Adverse Events (irAEs) Induced by Immune Checkpoint Inhibitors

**DOI:** 10.3390/curroncol30070498

**Published:** 2023-07-18

**Authors:** Yi-Shan Teng, Sebastian Yu

**Affiliations:** 1 Department of Dermatology, Kaohsiung Medical University Hospital, Kaohsiung Medical University, Kaohsiung 807378, Taiwan; chloet1202@gmail.com; 2 Department of Dermatology, School of Medicine, College of Medicine, Kaohsiung Medical University, Kaohsiung 807378, Taiwan; 3 Neuroscience Research Center, Kaohsiung Medical University, Kaohsiung 807378, Taiwan

**Keywords:** immune checkpoint inhibitor, immune-related adverse event, cutaneous immune-related adverse event, anti-CTLA-4 inhibitor, anti-PD-1 inhibitor, anti-PD-L1 inhibitor, anti-LAG-3 inhibitor

## Abstract

Over the past few decades, immune checkpoint inhibitors (ICIs) have emerged as promising therapeutic options for the treatment of various cancers. These novel treatments effectively target key mediators of immune checkpoint pathways. Currently, ICIs primarily consist of monoclonal antibodies that specifically block cytotoxic T-lymphocyte antigen 4 (CTLA-4), programmed cell death 1 (PD-1), programmed cell death-ligand 1 (PD-L1), and lymphocyte activation gene 3 protein (LAG-3). Despite the notable efficacy of ICIs in cancer treatment, they can also trigger immune-related adverse events (irAEs), which present as autoimmune-like or inflammatory conditions. IrAEs have the potential to affect multiple organ systems, with cutaneous toxicities being the most commonly observed. Although cutaneous irAEs are typically of low-grade severity and can usually be managed effectively, there are cases where severe irAEs can become life-threatening. Therefore, early recognition and a comprehensive understanding of the mechanisms underlying cutaneous irAEs are crucial for improving clinical outcomes in cancer patients. However, the precise pathogenesis of cutaneous irAEs remains unclear. This review focuses on the skin manifestations induced by ICIs, the prognosis related to cutaneous irAEs, and the exploration of potential mechanisms involved in cutaneous irAEs.

## 1. Introduction

Immune-checkpoint inhibitors (ICIs) have revolutionized the management of advanced malignancies over the past decade by offering groundbreaking therapeutic options [1,2]. Current biologic agents target natural immune checkpoint molecules, including cytotoxic T-lymphocyte antigen 4 (CTLA-4) [3,4], programmed cell death 1 (PD-1), programmed cell death-ligand 1 (PD-L1) [5,6], and lymphocyte activation gene 3 protein (LAG-3) [7], to enhance their antitumoral activity. While immune-checkpoint inhibitors hold great promise, their non-specific immunologic activations can give rise to various autoimmune-like or inflammatory diseases known as immune-related adverse events (irAEs) [8,9]. These adverse events predominantly affect the skin, gastrointestinal tract, liver, and endocrine glands, but they have the potential to involve any organ system [10,11].

Cutaneous immune-related adverse events (irAEs) are the most common complications associated with ICIs and often present as the initial manifestation. These events usually develop within the first few weeks to months after initiating immunotherapy, but they can occur at any time, even after treatment discontinuation [12]. A wide range of clinical presentations, including morbilliform eruptions, pruritus, lichenoid eruptions, psoriasiform dermatitis, vitiligo, bullous disorders, alopecia, and severe cutaneous adverse reactions (SCARs), have been reported [8,10,11,13,14]. These adverse events can significantly impact patients’ quality of life and may require the discontinuation of treatment. Therefore, early recognition and prompt management of cutaneous irAEs is important to minimize the immunotherapy-related morbidity and to achieve favorable outcomes in cancer patients. In this review, we focus on the clinical presentations and the possible mechanisms of various cutaneous irAEs.

## 2. Search Strategy

We systematically searched PubMed to identify original articles reporting the results of ICI-induced cutaneous irAEs. The search strategy was based on the following combinations of free text keywords and Medical Subject Heading (MeSH) terms: “immune-related adverse event(s)”, “cutaneous immune-related adverse event(s)”, “irAE(s)”, “cutaneous irAE(s)”, “mechanism”, “pathogenesis”. The Boolean operators used were “AND” and “OR”. We also included the names of specific ICIs in our search terms. In addition to the identified articles, we reviewed the references of clinical trials, review articles, observational studies, and animal experimental articles that discussed the mechanism of cutaneous irAEs. Articles written in languages other than English or those evaluating different forms of irAEs were excluded from our analysis.

## 3. The Biological Function of Immune Checkpoints

Immune checkpoint molecules are negative regulators that play an important role in maintaining immune homeostasis and downregulating immune responses to prevent autoimmunity. However, tumor cells can seize control of this system to evade the host immune system by activating the immune checkpoints to suppress immune responses. As a result, the blockade of immune checkpoint pathways disrupts these inhibitory signals, leading to immune activation, the induction of antitumoral immunity, and offering potential therapeutic benefits for patients with cancer. Currently, the Food and Drug Administration has approved several immune checkpoint inhibitors to treat various types of cancer. These biologic agents are monoclonal antibodies that primarily target specific molecules, including cytotoxic T-lymphocyte antigen 4 (CTLA-4) (ex: Ipilimumab) [15,16,17], programmed cell death 1 (PD-1) (ex: Cemiplimab, Nivolumab, Pembrolizumab, Dostarlimab) [7,15,16,17,18,19], programmed cell death-ligand 1 (PD-L1) (ex: Atezolizumab, Avelumab, Durvalumab) [20], and lymphocyte activation gene 3 protein (LAG-3) (ex: Relatlimab) [7]. Table 1 provides a list of current immune checkpoint inhibitors that are used for treating cutaneous malignancies (Table 1).

CTLA-4 (CD152) is a CD28 homolog mainly expressed by activated T cells and constitutively expressed on regulatory T cells (Treg cells). Its ligands, CD80 and CD86, are primarily located on the surface of antigen-presenting cells (APCs). They can bind either CTLA-4 or CD28, resulting in a co-inhibitory or co-stimulatory immune response, respectively. Although both receptors bind CD80 and CD86, CTLA-4 exhibits a much higher affinity than CD28 [21]. As a result, it effectively attenuates T cell activation by competing with the co-stimulatory molecule CD28 for binding to CD80 and CD86 [22,23,24]. By limiting CD28-mediated stimulatory signaling, CTLA-4 can decrease immune responses to weak antigens, such as self- or tumor antigens. Additionally, intrinsic CTLA-4 expression in Treg cells downregulates immune responses to maintain self-tolerance by inhibiting effector T cell proliferation and cytokine release [25,26]. Therefore, the loss of CTLA-4 may result in massive lymphoproliferation, profound T cell-mediated autoimmunity, and fatal multiorgan tissue destruction [3,27,28].

PD-1 (CD279) is expressed by activated T cells, B cells, natural killer (NK) cells, and some myeloid cells. It contributes to peripheral tolerance by binding to its ligands, PD-L1 (B7-H1; CD274) or PD-L2 (B7-DC; CD273), which are typically expressed on APCs, including macrophages, dendritic cells (DCs), and certain B cells. Without the PD-1 signaling, overactivation of immunity can lead to excessive immune-mediated tissue damage for the host. Although PD-L1 and PD-L2 share 38% identity, they exhibit distinct expression patterns. PD-L1 expression is prominently induced in APCs by interferon (IFN) and interleukin (IL)-17, whereas PD-L2 expression is stimulated by IL-4, suggesting they may play different roles in the immune response. Furthermore, both PD-1 and PD-L1 can be expressed by certain types of cancer cells and tumor-infiltrating immune cells, and this expression allows malignant cells to establish an immunosuppressive microenvironment and evade the host’s antitumor immunity [6,21,29,30].

LAG-3 (CD223) belongs to a novel class of immune checkpoint molecules and is widely expressed on various immune cells, including activated T cells, Treg cells, NK cells, B cells, and DCs. Through its interaction with its ligand, LAG-3 can negatively regulate T cell function and positively induce Treg cell activation, thereby contributing to the homeostasis of the immune system. Major histocompatibility complex II (MHC II), which is a ligand for CD4, also serves as the primary ligand for LAG-3. Notably, LAG-3 shows much higher affinity to MHC-II than CD4, suggesting that it may downregulate the T cell function via competition with CD4. In addition, several potential ligands of LAG-3, such as galactose lectin-3 (Galectin-3) [31,32], fibrinogen-like protein 1 (FGL1) [33], and hepatic sinusoid endothelial cell lectin (LSECtin) [34,35], have been reported in previous studies. These ligands are present on diverse cell types within the tumor microenvironment (TME), and their interaction with LAG-3 can facilitate tumor immune evasion by dampening anti-cancer immune responses [36,37,38,39,40].

## 4. Cutaneous Immune-Related Adverse Events

Despite the remarkable therapeutic efficacy of immune checkpoint inhibitors, their usage can give rise to a wide array of autoimmune and autoinflammatory reactions referred to as irAEs, due to their non-specific activation of the immune system. Among these irAEs, cutaneous irAEs are the most prevalent and often present as the earliest symptoms in patients undergoing immunotherapy. Generally, cutaneous irAEs emerge within weeks to months after initiating treatment with immune checkpoint inhibitors, although they can occur at any time, even after discontinuation of treatment [12]. Most cutaneous toxicities are mild to moderate in severity (CTCAE grades 1–2) and typically resolve spontaneously. In rare cases, severe cutaneous adverse reactions (SCARs) can occur, including conditions like Stevens-Johnson syndrome (SJS), toxic epidermal necrolysis (TEN), drug reaction with eosinophilia and systemic symptoms (DRESS), and acute generalized exanthematous pustulosis (AGEP) [41,42,43].

The occurrence of cutaneous irAEs has been reported in 30% to 60% of patients receiving treatment. Among various ICIs, anti-CTLA-4 monotherapy has a significantly higher rate of cutaneous irAEs (44–59%) compared to anti-PD-1 (34–42%) and anti-PD-L1 monotherapy (20%). Furthermore, the combination of CTLA-4 and PD-1 inhibition generally leads to an increased incidence (59–72%) and severity of skin toxicities [43,44,45]. It is believed that cutaneous irAEs are not dependent on the dosage of ICIs and can occur regardless of the underlying malignancy. However, recent research suggests that melanoma and renal cell carcinoma may carry a higher risk compared to other types of cancer [45]. Additionally, patients with pre-existing autoimmune diseases or pre-existing skin damage are more prone to experiencing cutaneous irAEs [11,46]

## 5. Subtypes and Possible Mechanisms of Cutaneous Immune-Related Adverse Events

The precise mechanisms responsible for ICI-induced cutaneous irAEs are not fully understood. However, several potential pathogeneses have been proposed. These include the involvement of type IV hypersensitivity reactions, genetic variations in certain human leukocyte antigen (HLA) variants, activation of self-reactive T cells and B cells that target shared antigens found in both tumor cells and normal tissues, stimulation of B cells and the humoral immune response, increased production of proinflammatory cytokines with immune-related consequences, potential exposure of host antigens from tumor cells due to cytotoxic attacks, and potential exacerbation of drug eruptions due to concurrent medication usage. We have summarized the possible mechanisms of cutaneous irAEs in Table 2. 

### 5.1. Morbilliform (Maculopapular) Eruption

The morbilliform eruption is the most commonly observed cutaneous irAE. It affects approximately 49–68% of patients undergoing anti-CTLA-4 therapy and 20% of patients receiving PD-1/PD-L1 inhibitors [13,47]. Typically, the morbilliform rash occurs within the first three to six weeks after initiating ICIs treatment [10,12,48,49]. Clinical presentations include pruritic faint erythematous macules and papules that coalesce into plaques. It primarily affects the trunk and the extensor side of the extremities, while the head, palms, and soles are usually unaffected [13,14]. Histopathological examinations reveal interface changes and perivascular or periadnexal lymphocytic infiltration, with or without eosinophils. Although maculopapular rashes are usually mild and resolve on their own, they can occasionally serve as an initial presentation of severe cutaneous adverse reactions like SSJS/TEN or DRESS.

The detailed mechanisms of ICI-induced morbilliform eruption remains unclear, but there is a hypothesis suggesting an association with type IVc hypersensitivity reactions [12,50], in which cytotoxic T cells act as effector cells. Under immunotherapy, activated cytotoxic T lymphocytes can directly harm target cells by releasing cytotoxic cytokines, including perforin, granulysin, either granzymes or granzymes B, and through physical interaction via the FasL/FasR pathway. Moreover, type IVc hypersensitivity is also predominantly implicated in SJS/TEN [50,51].

### 5.2. Pruritus

Pruritus is another common cutaneous irAE associated with ICIs. It can occur in conjunction with other skin manifestations or as an isolated symptom. Studies have indicated that pruritus affects approximately 14% to 47% of patients receiving ICIs, with higher incidence rates seen in those receiving anti-CTLA-4 monotherapy (25–36%) and combination therapy (33–47%) [12,43]. Typically, pruritus manifests within one to twenty-seven weeks after starting therapy and is commonly observed on the scalp and trunk, while the face, anterior neck, genitalia, and soles are usually unaffected [11,52,53]. In most cases, pruritus induced by ICIs is of low severity, and less than 2% of patients develop refractory high-grade pruritus [54,55].

The most extensively studied cytokines in the pathogenesis of itch are Th2 cytokines, including IL-4, IL-13, and IL-31. Certain Th1 cytokines, such as IL-17, may also be involved in psoriasis-related itch. Therefore, pruritus induced by ICIs may result in Th1/Th2 dysregulation, leading to the production of cytokines that promote skin inflammation [56,57]. However, the precise mechanism of ICI-related pruritus is not yet fully understood.

Genetic differences in human leukocyte antigen (HLA) haplotypes have been implicated in predisposing patients to develop irAEs. As a result, it is believed that genetic factors play a role in the pathogenesis of irAEs. Some irAEs closely resemble established autoimmune disorders that are associated with specific HLA risk alleles. For example, ICI-related colitis has been linked to HLA-DQB1*03:01 [58,59], ICI-induced hypothyroidism to HLA-DR8 [60], and ICI-related arthritis to HLA-DRB1*04:05 [61]. Similarly, specific HLA alleles have been found to be associated with ICI-induced pruritus, such as HLA-DRB1*11:01, as reported in several studies [59,62]. Despite extensive investigations, the exact mechanism linking HLA variants to irAEs remains elusive.

### 5.3. Lichenoid Eruptions

Lichenoid eruptions are more prevalent in patients receiving PD-1/PD-L1 inhibitors compared to those receiving CTLA-4 inhibitors [13,47,63,64,65,66]. These eruptions usually occurs between six to twelve weeks after initiation of ICI therapy [11,12], affecting less than 17% of patients using anti-PD-1 agents [67]. Clinical manifestations of lichen planus (LP) are characterized by shiny, flat-topped, polygonal, erythematous to violaceous papules and plaques with a thin, white lacelike pattern (Wickham striae) on the surface of the lesions, and it often exhibits symmetrical distribution on the flexural areas of the extremities. In contrast, ICI-associated lichenoid eruptions are pruritic, multiple, often grouped and confluent, purple-colored, flat-topped, slightly keratotic papules, or plaques mostly on the extensor surfaces of extremities and the trunk. Moreover, unlike lichen planus, ICI-induced LP-like eruptions are less frequently involved in the genital or oral mucosa, and Wickham striae are usually absent [13,68,69,70,71,72,73,74,75]. Both lichen planus (LP) and lichenoid eruptions share common histopathological findings, such as interface changes characterized by a dense band-like superficial infiltration of lymphocytes, a saw-tooth rete ridge pattern, hyperkeratosis, hypergranulosis, and the presence of apoptotic cells in the basal layer of the epidermis. However, in ICI-induced LP-like eruptions, there are additional distinctive features, including focal spongiosis, parakeratosis, focal interruption of the granular layer, and the presence of eosinophils or necrotic keratinocytes [13,68,69]. Furthermore, rare variants of LP, including ulcerative LP [70], hyperkeratotic LP [71], inverse LP [72], bullous LP [73,74], and lichen nitidus [75], have also been reported in association with ICIs. The detailed pathogenesis of ICI-induced lichenoid eruption remains unclear. However, it is believed that blocking the PD-1/PD-L1 pathway may enhance inflammatory reactions by activating the immune system, such as cytotoxic T cells and APCs. Prominent interferon-γ (IFN-γ) production in patients with oral LP receiving PD-1 inhibitors was found in a previous report [76]. Another study revealed increased mRNA expression of IFN-γ and granzyme B after anti-PD-1 agent treatment [77]. In addition, the expression of PD-L1 in keratinocytes has been speculated to play a protective role against cytotoxic T cells in a murine model. Furthermore, MHC-class-II molecule expression on keratinocytes induced by IFN-γ was also noted in a previous paper [78]. Based on these findings, it is hypothesized that inhibition of immune checkpoints may facilitate antigen presentation and lead to damage of basal epidermal keratinocytes through the activation of autoreactive cytotoxic T cells, which are mediated by IFN-γ and other molecules [79].

Gene expression profiles and immune compositions of ICI-related lichenoid eruption have also been analyzed previously. One study reported increased CD14+ and CD16+ monocytes and upregulation of toll-like receptor 2 (TLR 2) and TLR4 in patients with ICI-induced lichenoid dermatitis. Additionally, this study also found higher numbers of T-Bet+ (Th1) cells and lower numbers of Gata-3+ (Th2) cells and FoxP3 (T regulatory) cells in the immune profiles of lichenoid eruptions. These findings suggest that the innate immune response may also be involved in the initiation of lichenoid eruptions through the CD14/TLR signaling pathway [80].

### 5.4. Psoriasiform Eruptions

Psoriasiform eruptions are more frequently observed in patients receiving PD-1/PD-L1 inhibitors than in patients receiving CTLA-4 inhibitors. Approximately 3–12% of patients treated with PD-1 inhibitors may be affected [13,14,81,82]. These eruptions can be categorized into two types, including newly onset psoriasis (de novo psoriasis) and a flare-up of pre-existing psoriasis (reactivated psoriasis). A previous report revealed that out of 115 cases of ICI-induced psoriasis, 70% of patients developed de novo psoriasis, while 30% had a history of psoriasis [83]. The lesions commonly appear after three to twelve weeks of treatment [10,12], with an average onset time of about fifty days for psoriasis flare-ups and about ninety-one days for newly developed psoriasis [81]. The most common type of ICI-induced psoriasiform eruption is plaque psoriasis, characterized by erythematous silvery scaly plaques with well-defined borders localized on the extensor surfaces of extremities and the trunk. Less frequent variants, including palmoplantar, pustular, guttate, and inverse psoriasis, have also been reported previously [83]. Histopathological findings resemble typical psoriasis vulgaris, with features such as parakeratosis, hypogranulosis, acanthosis with elongated rete ridges, and a perivascular lymphocytic infiltration [10,13,63]. Moreover, compared to typical psoriasis, variable spongiosis, infiltration of eosinophils, and lichenoid change can also be found in ICI-related psoriasis [84,85].

In the general population, psoriasis is mainly driven by the activation of the Th17 pathway, and several cytokines, including tumor necrosis factor alpha (TNF-α), IL-23, IL-17, and IL22, play important roles in its pathogenesis [86]. The detailed mechanisms underlying ICI-induced psoriasiform eruptions are still unclear. However, studies in animal models have shown that blocking immune checkpoint molecules can result in enhanced production of IL-17A and IL-22 via activated T cells, either through gene silencing or monoclonal antibody treatment. Based on these findings, it is hypothesized that ICIs may lead to the overproduction of proinflammatory cytokines, mediated by activated Th17 cells, which in turn promote neutrophil recruitment and keratinocyte hyperproliferation, ultimately exacerbating or inducing psoriasis.

In patients, psoriasis is frequently associated with obesity and imbalanced dietary habits [87]. In a murine model system, it has been observed that the consumption of a Western diet (WD) high in fat and simple sugars dramatically increases the expression of PD-1 on γδ low (GDL) T cells, which are the main producers of IL-17A, thereby exacerbating the psoriasiform dermatitis induced by imiquimod (IMQ). Additionally, mice fed a WD and exhibiting obesity demonstrate a greater severity of IMQ-induced psoriasiform dermatitis compared to control mice when administered anti-PD1 treatment. Based on these findings, it is hypothesized that WD-induced obesity may be involved in the development of de novo psoriasis-like skin lesions or the worsening of pre-existing psoriasis in patients undergoing anti-PD-1 therapy, although the exact mechanism remains unknown [88,89].

### 5.5. Vitiligo-like Depigmentation

Vitiligo is an autoimmune disease characterized by the presence of well-defined, depigmented macules or patches resulting from the loss of functional melanocytes in the epidermis [90,91,92]. Among the various types of cutaneous irAEs, vitiligo-like depigmentation (VLD) is a subtype that occurs as a result of reactive autoimmunity targeting melanocytes in normal tissues during immunotherapy. VLD is more frequently observed in patients with advanced melanoma, while its occurrence in other malignancies is relatively rare [91,93,94,95]. Compared to anti-CTLA-4 therapy, VLD is more often induced during anti-PD-1 therapy, with incidence rates ranging from 7–11% and 2–9%, respectively. Moreover, the lesions commonly develop between seven and sixty-five weeks after starting ICI therapy, with a median onset time of approximately twenty-six weeks, and these lesions often persist even after discontinuation of immunotherapy [11,12,96]. Despite the significant psychosocial impact, the occurrence of VLD during ICI therapy is significantly associated with a favorable prognosis in patients with melanoma, including prolonged progression-free survival and overall survival rates [97]. This correlation highlights the strong relationship between irAEs and enhanced antitumor activity [10,90,91,97].

The clinical manifestations of ICI-induced VLD differ from those of idiopathic vitiligo, which tends to occur more commonly in periorificial areas and acral surfaces. VLD is characterized by the presence of multiple flecked macules that evolve into larger patches, predominantly affecting sun-exposed areas in a symmetrical pattern. Unlike idiopathic vitiligo, VLD is not associated with the Koebner phenomenon [98]. Moreover, an inflammatory phase may precede the development of depigmented lesions in VLD [53,91], and depigmentation of the skin may coincide with poliosis of the eyelashes and scalp hair [43,53]. Histologically, VLD shows an inflammatory infiltrate in the dermis primarily composed of T cells and a lack of melanocytes.

The exact pathogenesis of immunotherapy-induced VLD remains uncertain, but several studies have proposed that its development is related to cross-reactivity against shared antigens present on both melanocytes and tumor cells. Upon treatment with anti-PD-1 agents, skin depigmentation occurs as a result of cytotoxic T cell activation against these shared antigens, which include Melanoma antigen recognized by T cells 1 (MART-1, also called Melan-A), GP100, tyrosinase-related proteins 1 and 2 (TRP1 and TRP2), and tyrosinase [97,99]. Immunohistochemical staining analysis has further revealed the presence of CD4 and MART-1/Melan-A specific CD8 T cells in close proximity to apoptotic melanocytes, indicating that anti-CTLA-4 antibodies may also contribute to stimulating an immune reaction against melanocytes [100].

### 5.6. Autoimmune Bullous Disease

Compared to other cutaneous irAEs, autoimmune bullous disorders are relatively less frequently observed. The incidence of ICI-induced bullous diseases is approximately 1% in patients treated with PD-1/PD-L1 inhibitors [12,13,101], and the onset time is typically between thirteen and eighty weeks after initiating immunotherapy, which is longer than other cutaneous irAEs [12,102,103]. Among the bullous diseases associated with immunotherapy, bullous pemphigoid (BP) is the most common presentation, followed by bullous lichenoid dermatitis and linear IgA bullous dermatosis [101]. The clinical presentations of ICI-induced BP resemble classic BP, characterized by a pruritic prodromal non-bullous phase followed by the development of tense bullae filled with serous or hemorrhagic fluid mainly localized on the trunk and extremities. Other variants, such as urticarial-predominant, eczematous rash, Grover disease–like, and dyshidrosiform, have also been reported previously [53,104]. However, unlike idiopathic BP, mucous membrane involvement is more common, with a frequency of up to 40% [64,105]. Histopathological findings are similar to those of spontaneous BP, which show subepidermal blisters with numerous eosinophils, and direct immunofluorescence reveals linear deposition of complement component 3 (C3) and immunoglobulin G (IgG) along the basement membrane zone [10,13,106].

It is believed that autoimmune bullous disorders induced by PD-1/PD-L1 inhibitors involve both T cell and B cell dysregulation. In T cell-independent humoral immunity, PD-1/PD-L1 blockades can enhance B-cell activation, leading to the production of disease-specific autoantibodies. These autoantibodies are responsible for cross-reactive immunogenicity against basement membrane proteins BP180 and BP230, which are expressed on certain cancer cells (such as melanoma and non-small cell lung carcinoma) as well as normal skin, thereby inducing BP [68,106,107,108]. In T cell-dependent humoral immunity, PD-1 acts as an activator for B cells interacting with either follicular helper T cells (Tfh) or follicular regulatory T cells (Tfr) within the germinal centers. Tfh cells play a role in the selection and survival of B cells, enabling their differentiation into memory B cells or high-affinity antibody-producing plasma cells. On the other hand, Tfr cells maintain immune balance by suppressing both Tfh cells and B cells. Inhibition of PD-1 may reduce the suppressive ability of Tfr cells and lead to potentially mutated B cells selected by Tfh cells. This can result in an aberrant production of low-affinity plasma cells, which contribute to the development of numerous antibody-mediated autoimmune disorders, including BP [107]. In addition to cross-reactivity, the production of autoantibodies against different epitopes, which is termed epitope-spreading phenomena, has also been observed in anti-PD-1/PD-L1-associated BP [107].

It has been reported that host genetic background may also be important in their susceptibility to irAEs, and several genetic variants associated with irAEs have been identified recently [109]. One SNP associated with an increased risk of autoimmune bullous pemphigoid has been linked to the DSC2 gene [110].

### 5.7. Stevens-Johnson Syndrome/Toxic Epidermal Necrolysis-like Reaction

ICI-related SCARs, such as SJS/TEN-like reactions, are rare dermatologic toxicities, but they can be potentially life-threatening [111,112,113,114]. The onset time of SCARs varies from one to twenty weeks after initiation of immunotherapy [12,115], and the mortality rates are approximately 10% for SJS, 30% for SJS-TEN overlap syndrome, and 50% for TEN, respectively [105,115,116]. The clinical presentations are similar to classical SJS/TEN, including fever, conjunctival injection, and malaise, followed by diffuse erythema, with or without targetoid lesions on the trunk and extremities, progressing to flaccid bullae with a positive Nikolsky sign. In addition, mucosal involvement of the conjunctivae, oral cavity, gastrointestinal tract, respiratory tract, and genitalia may be observed [46,117]. However, unlike classical SJS/TEN, the SJS/TEN-like eruption has a longer onset time and the symptoms are usually milder than typical SJS/TEN, with less ocular involvement and less denuded skin. It has also been found that a morbilliform eruption may precede the ICI-induced SJS/TEN-like reactions. Therefore, it is important to closely monitor patients with a morbilliform rash for red-flag signs, such as blister formation, a positive Nikolsky sign, development of targetoid lesions, skin pain or mucosal involvement [118]. Histopathological findings show full-thickness epidermal necrolysis with extensive keratinocyte necrosis, subepidermal bullae, and a varied degree of inflammation in the superficial dermis with a sparse dermal infiltrate.

Some studies speculate that the inhibition of checkpoint molecules interferes with the balance between the peripheral tolerance and the function of keratinocytes in protecting against damages, which eventually explains the frequent ICI-induced cutaneous eruptions. It has been found that PD-L1 is usually undetectable in normal skin, but ICI treatment can stimulate PD-L1 expression, triggering apoptosis of PD-L1 expressing keratinocytes induced by activated cytotoxic T cells [12,119]. Moreover, ICI-induced SJS/TEN-like eruptions exhibit a similar gene expression profile to classical SJS/TEN, both of which show increased expression of inflammatory chemokines, cytotoxic mediators (such as perforin and granzyme B), and apoptosis-promoting molecules (such as Fas Ligand). It is believed that, similar to morbilliform rash, type IVc hypersensitivity may also be involved in SJS/TEN-like reactions [12,115,120]. Furthermore, the enhancement of co-stimulatory factors and dysfunction of Treg cells are also associated with SJS/TEN pathogenesis [12].

## 6. Prognosis Related to Cutaneous irAEs

Cutaneous irAEs can indicate a favorable response to cancer treatment, signifying the activation of the immune system by ICIs to identify and attack cancer cells. Patients who encounter certain cutaneous irAEs are more likely to have an improved prognosis [121,122]. The strongest evidence supporting a positive prognosis is observed in cases of ICI-induced VLD during melanoma treatment, which is strongly correlated with improved, progression-free, and overall survival [92]. Additionally, other cutaneous reactions, including morbilliform exanthems, lichenoid and eczematous presentations, and immunobullous disorders, may also be linked to a better prognosis, although the evidence for these associations is not as firmly established [106,121,123,124]. Further investigations are necessary to determine the prognostic implications of different types of rashes and identify which patients are most susceptible to specific cutaneous irAEs.

## 7. Conclusions

ICIs are being recognized as promising therapeutic approaches for managing advanced malignancies. With the increasing use of immunotherapy, there is a simultaneous rise in the incidence of irAEs associated with these treatments. These irAEs share common characteristics, including immune cell dysregulation and potential genetic predisposition. They often present as inflammatory or autoimmune reactions resulting from the dysregulation of the immune response. IrAEs have the potential to affect multiple organ systems, with cutaneous toxicities being the most commonly observed. While most cases of cutaneous irAEs are mild and can be managed effectively, there are rare instances where severe irAEs can be potentially life-threatening and require prompt treatment, often leading to discontinuation of ICIs therapy. Therefore, it is crucial to accurately diagnose, promptly manage, and comprehend the pathogenesis of cutaneous irAEs in order to minimize adverse effects and achieve favorable outcomes.

The mechanisms underlying ICI-induced cutaneous irAEs remain unknown. It has been hypothesized that these adverse events may involve the activation of self-reactive T cells targeting common antigens, stimulation of B cells and humoral immunity, increased release of proinflammatory cytokines, and the occurrence of type IV hypersensitivity reactions. Recent studies have also suggested that the host’s genetic background may play a role in determining susceptibility to irAEs. In the future, it will be important to identify and validate predictive biomarkers, potentially through genome profiling, to better identify patients at risk of developing irAEs and improve their medical management. Additionally, careful monitoring of patients’ history of skin disorders will be essential prior to initiating ICI treatment.Furthermore, in light of recent research, an in-depth evaluation of cutaneous irAEs can offer valuable insights into patient prognosis, biomarkers for predicting future irAEs, and associated risk factors. Given the potential impact of cutaneous irAEs on treatment outcomes, there is a compelling need for continued exploration of approaches that incorporate cutaneous irAEs into patient stratification and treatment strategies. This may involve integrating patient-specific factors such as genetic markers, baseline immune profiles, or other biomarkers that can aid in predicting the likelihood of cutaneous irAE occurrence. Consequently, dermatologists should ensure that they have a comprehensive understanding of diverse cutaneous manifestations, irrespective of their prevalence, as well as the treatment approaches and underlying mechanisms involved in effectively managing cutaneous irAEs.

## Figures and Tables

**Table 1:** FDA-Approved Immune Checkpoint Inhibitors for Cutaneous Malignancies.

Immune Checkpoint Inhibitors	Target	Indications of Cutaneous Malignancy	FDA Approval
Ipilimumab (Yervoy)	CTLA-4	Metastatic or unresectable melanoma	2011
Nivolumab (Opdivo)	PD-1	Advanced melanoma	2014
Pembrolizumab (Keytruda)	PD-1	Advanced melanoma Advanced cutaneous SCC	2014 2020
Cemiplimab (Libtayo)	PD-1	Cutaneous SCC Locally advanced or metastatic BCC	2018 2021
Avelumab (Bavencio)	PD-L1	Metastatic MCC	2017
Relatlimab (Opdualag) combined with Nivolumab	LAG-3	Metastatic or unresectable melanoma	2022

Abbreviations: CTLA-4, cytotoxic T lymphocyte associated protein 4; FDA, Food and Drug Administration; LAG-3, lymphocyte activation gene 3 protein; PD-1, programmed cell death protein 1; PD-L1, programmed cell death 1 ligand 1; SCC, squamous cell carcinoma; BCC, basal cell carcinoma; MCC, Merkel cell carcinoma.

**Table 2:** Possible pathogenesis, onset time, and frequency of cutaneous irAEs.

Cutaneous irAEs	Onset Time	Mainly Associated ICIs	Frequency	Possible Mechanisms
Morbilliform eruption	3–6 weeks	Anti-CTLA-4 > Anti-PD-1/PD-L1	Anti-CTLA-4: 49–68% Anti-PD-1/PD-L1: 20%	Type IV hypersensitivity reaction
Pruritus	1–27 weeks	Anti-CTLA-4 > Anti-PD-1/PD-L1	Anti-CTLA-4: 25–36% Combination: 33–47%	Genetic difference in HLA variants Increased proinflammatory cytokines
Lichenoid eruption	6–12 weeks	Anti-PD-1/PD-L1	Anti-PD-1/PD-L1: <17%	Autoreactive T cells against common antigen Increased proinflammatory cytokines Innate immunity
Psoriasiform eruption	3–12 weeks	Anti-PD-1/PD-L1	Anti-PD-1/PD-L1: 3–12%	Th17 pathway activation Increased proinflammatory cytokines
Vitiligo like depigmentation	7–65 weeks	Anti-PD-1/PD-L1 > Anti-CTLA-4	Anti-PD-1/PD-L1: 7–11% Anti-CTLA-4: 2–9%	Autoreactive T cells against common antigen
Bullous pemphigoid	13–80 weeks	Anti-PD-1/PD-L1	Anti-PD-1/PD-L1: 1%	T cell dependent humoral immunity T cell independent humoral immunity
SJS/TEN	1–20 weeks	Anti-CTLA-4 > Anti-PD-1/PD-L1	Rare	Autoreactive T cells against common antigen Type IV hypersensitivity reaction

Abbreviations: irAEs, immune-related adverse events; SJS/TEN, Stevens–Johnson syndrome/toxic epidermal necrolysis; ICIs, immune checkpoint inhibitors; anti-CTLA-4, anti-cytotoxic T-lymphocyte antigen 4; anti-PD-1, anti-programmed cell death 1; anti-PD-L1, anti-programmed cell death-ligand 1; HLA, human leukocyte antigen.

## Data Availability

Not applicable.
